# The effects of elevated phosphate on the kidney - damaging the gatekeeper

**DOI:** 10.1007/s00424-026-03160-5

**Published:** 2026-03-30

**Authors:** Tanecia Mitchell, Vivek Verma, Abul Fajol, Christian Faul

**Affiliations:** 1 Department of Urology, Heersink School of Medicine, The University of Alabama at Birmingham, Birmingham, AL USA; 2 Section of Mineral Metabolism, Division of Nephrology, Department of Medicine, Heersink School of Medicine, The University of Alabama at Birmingham, Tinsley Harrison Tower 611L, 1720 2nd Avenue South, Birmingham, AL 35294 USA

**Keywords:** chronic kidney disease, hyperphosphaturia, nephrocalcinosis, nephrolithiasis, phosphate

## Abstract

The kidney is a major regulator of phosphate metabolism. The body can lower systemic phosphate levels by increasing renal phosphate excretion, and kidney injury results in elevated serum phosphate concentrations (hyperphosphatemia). Chronic kidney disease (CKD) is associated with various organ injuries, including vascular calcification and cardiovascular disease, where hyperphosphatemia acts as a pathologic driver. Here we discuss hyperphosphatemia not as a consequence of kidney disease but as a potential contributor to kidney damage. We describe how increases in renal tubular phosphate levels (hyperphosphaturia), rather than hyperphosphatemia contribute to kidney injury in CKD. Tubular phosphate can form microcrystals with calcium which damages renal epithelial cells, induces fibrosis and inflammation, and causes parenchymal calcification. Calcium phosphate microcrystals can grow and form larger deposits in the renal collecting system, and potentially contribute to the formation of kidney stones. Therefore, hyperphosphaturia might not only contribute to kidney damage in CKD, but could also cause kidney injury in genetic diseases with reduced renal phosphate uptake. Finally, since high dietary phosphate intake increases renal phosphate excretion, we discuss if prolonged phosphate loading in the absence of CKD can induce kidney damage. We propose that a better understanding of the pathologic actions of phosphate on the kidney will help to identify novel therapeutic strategies to prevent renal injury and disease progression in patients with CKD and in other renal conditions, such as kidney stone formation. Lowering dietary phosphate intake might not only have reno-protective effects in patients with pre-existing kidney damage but also in healthy individuals.

## Introduction

**Fig. 1:**
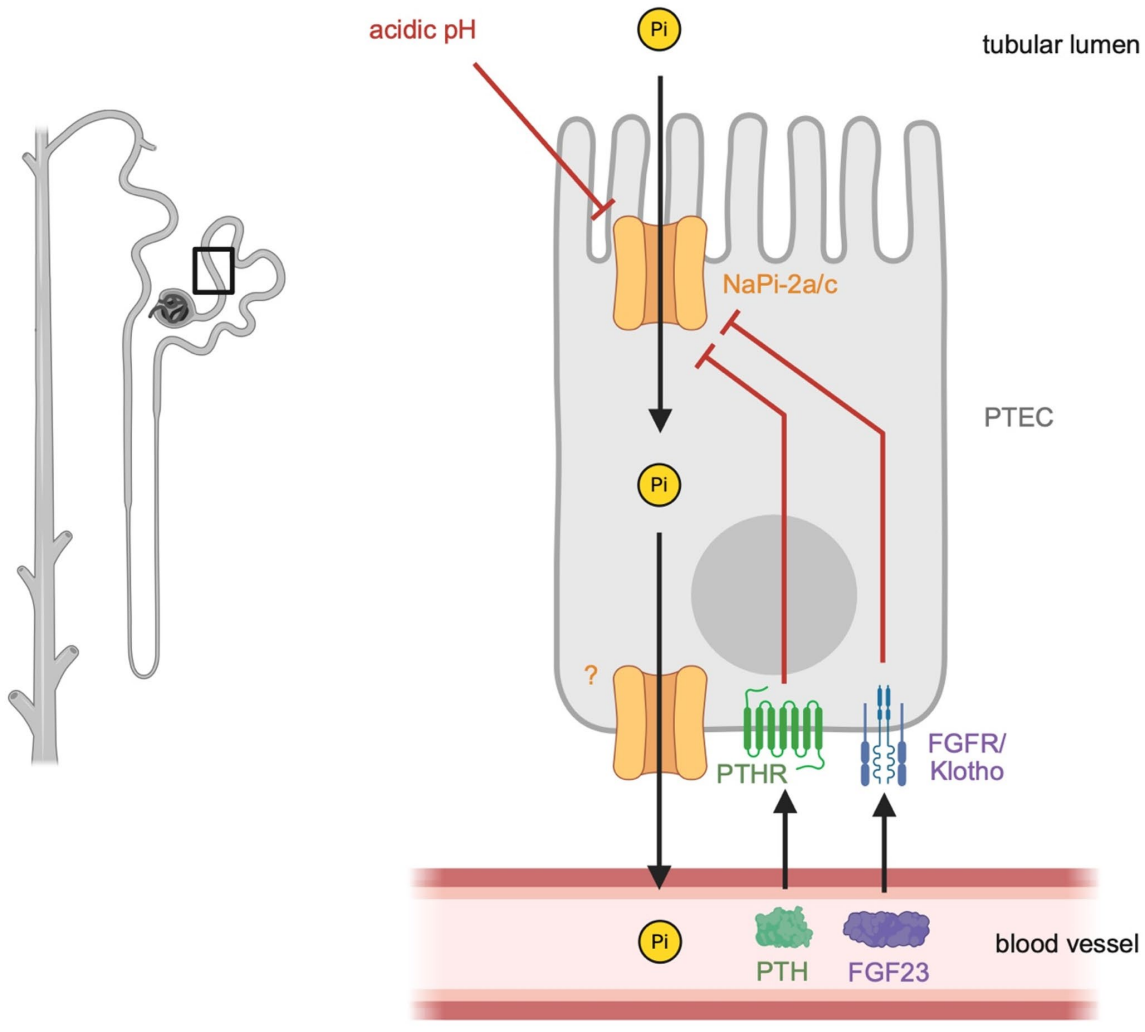
The regulation of phosphate uptake by the kidney Filtered phosphate (Pi) is taken up by proximal tubular epithelial cells (PTEC) on the apical side via sodium-dependent phosphate transporters NaPi-2a and NaPi-2c and released into the circulation on the basolateral side by a transport mechanism that is not well understood. Circulating hormones, fibroblast growth factor 23 (FGF23) and parathyroid hormone (PTH) bind FGF receptor (FGFR)/Klotho complexes and PTH receptors (PTHR), respectively, on the basolateral side and induce signaling events leading to a reduction of NaPi-2a/c levels on the apical cell surface. An acidic pH in the tubular lumen inhibits NaPi-2a/c activity. FGF23, PTH and an acidic pH reduce tubular phosphate uptake and thereby elevate tubular phosphate concentrations

Phosphorus is an essential element that is taken up with the diet in the form of negatively charged inorganic phosphate by the small intestine [1]. Phosphate is distributed via the blood to all cells which use phosphate for multiple cellular functions and structures [2]. Most of the body’s phosphate is stored in the extracellular matrix of bone as the calcium salt, hydroxyapatite, where it forms an important structural component of the skeleton and serves as a phosphate reservoir. The kidney can reabsorb filtered phosphate or excrete it with the urine, thereby serving as the body’s gatekeeper of phosphate balance [3]. Phosphate homeostasis requires the communication between the intestine, bone, and kidney [4, 5], which is mediated by fibroblast growth factor 23 (FGF23), parathyroid hormone (PTH), and active vitamin D (1,25D) [6, 7]. About 80% of the filtered phosphate is reabsorbed in the renal proximal tubule, which is mediated by secondary active sodium-dependent transport via sodium phosphate (NaPi) cotransporters [8]. NaPi-2a and NaPi-2c are highly expressed in the brush border of the proximal tubular epithelial cells and mediate the majority of renal phosphate uptake [3]. It appears that the rate of phosphate reabsorption is primarily regulated by the abundance of NaPi-2a and NaPi-2c expression at the cell surface [3]. High dietary phosphate intake causes increased excretion of phosphate in the urine (also called phosphaturia) by downregulating renal NaPi-2a/c expression, which is mainly mediated by circulating FGF23 and PTH and their receptors on proximal tubular epithelial cells, which are FGF receptor (FGFR)/klotho complexes and PTH receptors (PTHR), respectively (Fig. 1) [9].

While the key factors regulating renal phosphate metabolism are well established, novel aspects and nuances of phosphate physiology have been discovered more recently, suggesting that a more complex network of phosphate sensors and regulators exists. For example, it is known that a dietary phosphate load triggers the release of FGF23 from the bone [10, 11]. Two recent studies suggested that in this context, the kidney serves as a phosphate sensor that regulates FGF23 production [12, 13]. It appears that phosphate is the rate-limiting step for glycolysis in renal proximal tubular epithelial cells. Therefore, when renal phosphate filtration and absorption are elevated following dietary phosphate load, glycolysis in the proximal tubule is promoted resulting in the increased production of glycerol-3-phosphate (G-3-P). Subsequently, G-3-P is secreted into the circulation and targets the bone to stimulate FGF23 production, which then downregulates NaPi-2a and NaPi-2c expression in the proximal tubule and reduces renal phosphate uptake and the glycolytic flux. Thereby, renal G-3-P provides a negative feedback loop connecting increased phosphate uptake with increased FGF23 production [14]. Another recent experimental study suggests that the liver acts as a sensor for elevations in serum phosphate levels, at least in the acute postprandial phase, and quickly communicates with the kidney to lower circulating phosphate concentrations [15]. In this context, the crosstalk between the liver and the kidney is nerve-mediated and causes reductions in renal NaPi-2a levels and elevations in renal phosphate excretion independently of the endocrine regulators FGF23 and PTH.

Since the body can only release phosphate via renal excretion, impaired kidney function causes increases in serum phosphate levels (also called hyperphosphatemia), as observed in patients with chronic kidney disease (CKD) [7]. Here, we will discuss various studies indicating that hyperphosphatemia is not only the consequence of kidney injury but might also contribute to the progression of kidney damage. Phosphate can form crystals with calcium, which under pathologic conditions, such as in CKD, can result in the formation of calcium phosphate microcrystals outside of the skeletal system. This ectopic calcification (also called calcinosis) can occur in the vasculature as well as in extra-vascular tissue areas, thereby causing damage in various soft tissues [16]. Several experimental studies suggest that hyperphosphatemia is a major driver of vascular calcification, which might contribute to cardiovascular disease and high mortality rates in patients with CKD [17]. Ectopic calcifications also include nephrocalcinosis which is the appearance of calcium phosphate microcrystals in the renal tubules and parenchyma [18, 19]. Here, we will discuss whether increases in renal tubular phosphate levels (hyperphosphaturia) rather than high serum concentrations of phosphate (hyperphosphatemia) contribute to kidney damage in CKD. Furthermore, it is well established that calcium phosphate is a component of many kidney stones [20], and we will discuss whether hyperphosphaturia can promote the formation of larger calcium phosphate deposits in the renal collecting system and thereby potentially contribute to the formation of kidney stones (also called nephrolithiasis) [20]. Finally, since high dietary phosphate intake increases serum phosphate levels and renal phosphate excretion [21], we will discuss if prolonged elevations of phosphate in the absence of CKD can induce kidney damage.

## Hyperphosphaturia contributes to the progression of kidney injury in CKD

When the number of functional nephrons decline, as in CKD, individual nephrons increase phosphate excretion to prevent hyperphosphatemia. This concept is supported by early micropuncture studies conducted on nephrons isolated from a rat model of CKD which demonstrated that tubular phosphate concentrations were significantly elevated [22]. Moreover, studies in CKD patients have found that urinary fractional excretion of phosphate progressively increases with declining kidney function to maintain serum phosphate levels in the normal range, which is mediated by a rise in the serum levels of FGF23 and PTH [23, 24]. Therefore, compared to the urinary phosphate excretion, serum phosphate levels increase relatively late during the progression of CKD, when kidneys have failed and patients have reached end-stage renal disease (ESRD) [23]. Various clinical studies have reported associations between elevated serum phosphate concentrations and morbidity and mortality in patients with ESRD [25–32]. Furthermore, cell culture and animal studies have shown that high phosphate levels can directly induce vascular calcification, and clinical ESRD studies found strong associations between hyperphosphatemia and cardiovascular disease [33, 34]. Clinical studies also reported that serum phosphate levels are associated with and predict a faster progression of CKD [28, 35–39]. Furthermore, in ESRD patients receiving a kidney transplant, higher serum phosphate levels are associated with a significantly higher risk of transplant failure [40]. In various animal models of CKD, the development of renal injury is associated with the presence of hyperphosphatemia [41–50]. Therefore, it is possible that in CKD hyperphosphatemia does not only contribute to cardiovascular disease, but also promote the progression of kidney damage.

Experimental studies in rodents have shown that when renal mass and function are reduced, smaller quantities of dietary phosphate intake are required to induce kidney injury [51–53]. As shown in rats with reduced nephron number induced by subtotal nephrectomy, the administration of a high phosphate diet causes a spectrum of pathohistological changes in the remnant kidney, including tubular dilation, interstitial fibrosis, and inflammatory cell infiltration, which is accompanied by an accelerated reduction in kidney function [43–46, 51, 54–56]. Moreover, a high phosphate diet increased renal fibrosis in a mouse model with uninephrectomy and ischemia-reperfusion injury [57]. Similarly, in the Cy/+ rat model with a spontaneous genetic mutation that leads to polycystic kidney disease (PKD) and progressive CKD, the administration of a high phosphate diet promoted the progression of kidney injury [47]. In a different rat model of PKD, increased dietary phosphate intake exacerbated cyst formation and disease progression [58]. Overall, it appears that in rodents with pre-existing kidney injury increasing dietary phosphate load aggravates kidney damage. However, this effect might depend on the animal model, since a phosphate feeding study conducted in a rat model of CKD induced by an adenine-rich diet did not detect an association between dietary phosphate content and the severity of kidney injury [59]. Similarly, the increase of dietary phosphate in mice with adenine diet-induced CKD did not worsen kidney injury [60]. It is possible that in this particular model of CKD adenine might bind or interfere with dietary phosphate thereby inhibiting potential additive effects of CKD and high phosphate on the kidney [60].

Studies in partially nephrectomized rats on a high phosphate diet suggest that kidney injury can occur in the presence of hyperphosphaturia and in the absence of hyperphosphatemia [51, 55, 61], indicating that increased renal phosphate excretion rather than elevated serum phosphate concentrations are the culprit for the progression of kidney injury. This hypothesis is also supported by feeding studies in uninephrectomized rats where polyphosphate does not increase serum phosphate levels but causes more severe kidney injury when compared to feeding with high phosphate salts which induced hyperphosphatemia [56]. Similarly, a feeding study in Cy+ rats has shown that the administration of a grain-based diet with low phosphate bioavailability and a casein-based diet with high phosphate bioavailability has the same effect on serum phosphate levels, while only the later diet increases urinary phosphate excretion and kidney injury [47]. Furthermore, a diet with fermentable inulin fibers, which alters the gut microbiota, lowers serum phosphate levels in Cy/+ rats without reducing the progression of kidney injury [62]. Overall, the pathological actions of phosphate on the kidney during CKD seem to be driven by hyperphosphaturia and depend on the source and bioavailability of dietary phosphate [63, 64].

**Fig. 2:**
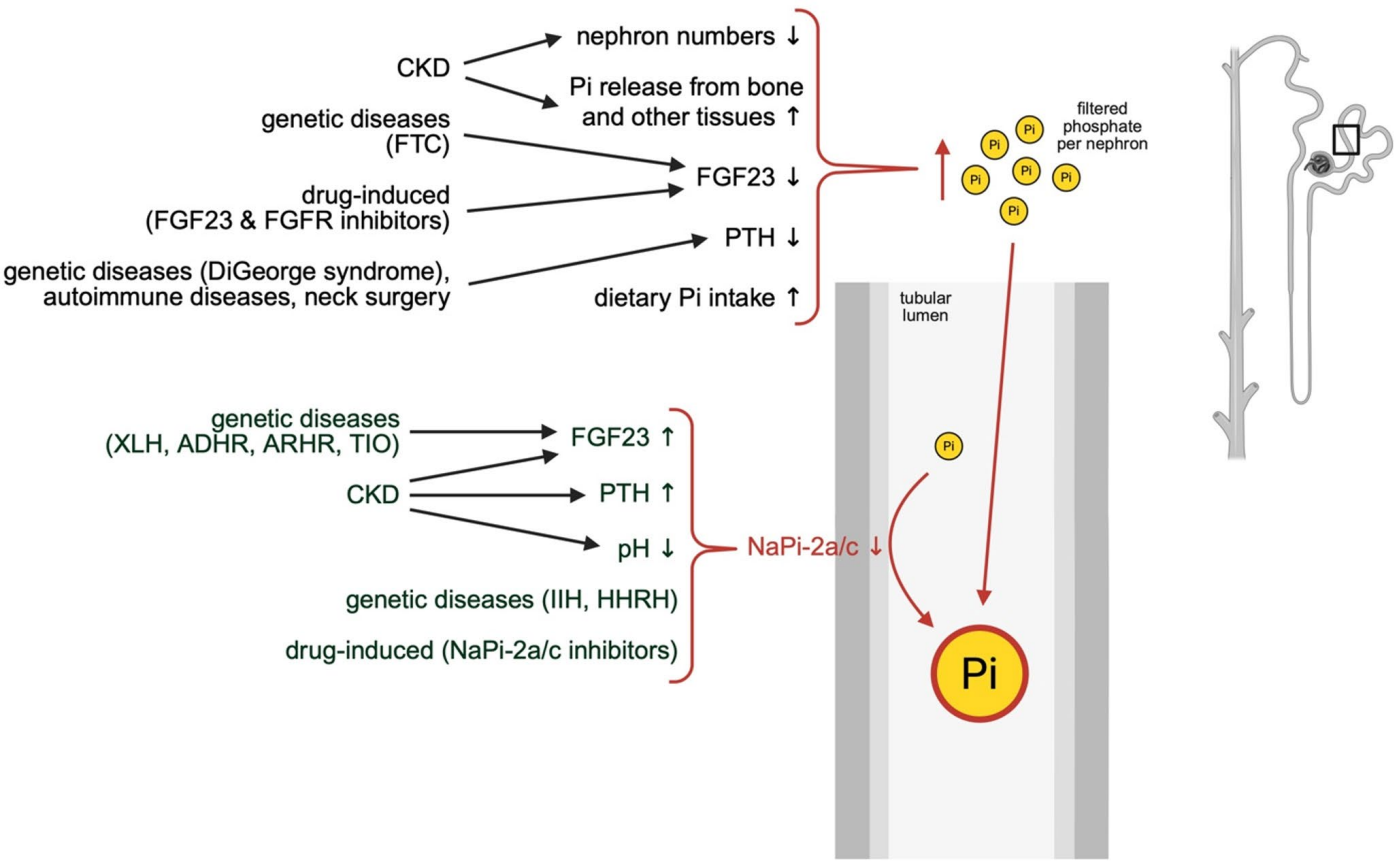
The development of hyperphosphaturia An increase in circulating phosphate (Pi) which is freely filtered and enters the tubular system as well as reductions in phosphate uptake by the proximal tubule can result in elevations in tubular phosphate levels. Therefore, scenarios that elevate serum phosphate levels and increase the phosphate load per nephron can cause hyperphosphaturia. This includes increased dietary Pi intake and Pi release from internal stores in chronic kidney disease (CKD). Similarly, scenarios that reduce the activity or expression of the sodium-dependent phosphate transporters NaPi-2a and NaPi-2c result in hyperphosphaturia. Hyperphosphatemia and hyperphosphaturia occur in CKD and in specific genetic diseases and they can be induced by drugs inhibiting fibroblast growth factor 23 (FGF23) and FGF receptor (FGFR) signaling or NaPi-2/c activity. ADHR: autosomal dominant hypophosphatemic rickets; ARHR: autosomal recessive hypophosphatemic rickets; CaPi: calcium phosphate; FTC: familial tumoral calcinosis; HHRH: hereditary hypophosphatemic rickets and hypercalcuria; IIH: idiopathic infantile hypercalcemia; PTH: parathyroid hormone; TIO: tumor-induced osteomalacia; XLH: X-linked hypophosphatemia

Collectively, these studies suggest that in CKD, an increase in phosphate excretion per nephron, rather than systemic changes in phosphate content induces kidney damage (Fig. 2). This hypothesis is supported by clinical studies in small cohorts of CKD patients which found that increased urinary phosphate excretion is associated with CKD progression [55, 65]. Moreover, a recent cross-sectional study in pre-dialysis patients found that increases in tubular phosphate levels are associated with tubular damage [66]. It is possible that proximal tubular epithelial cells are damaged when exposed to high levels of extracellular phosphate from the apical side, which is supported by cell culture studies showing that phosphate elevations induce senescence and a pro-fibrotic and pro-inflammatory response in renal epithelial cells [57, 67]. Overall, there is growing evidence that phosphaturia in the absence of hyperphosphatemia can promote kidney injury in CKD. However, unlike serum phosphate levels, increases in urinary phosphate excretion have not been consistently associated with cardiovascular events and mortality in large CKD cohorts [68, 69].

### Tubular calcium phosphate microcrystals contribute to the progression of kidney injury in CKD

**Fig. 3:**
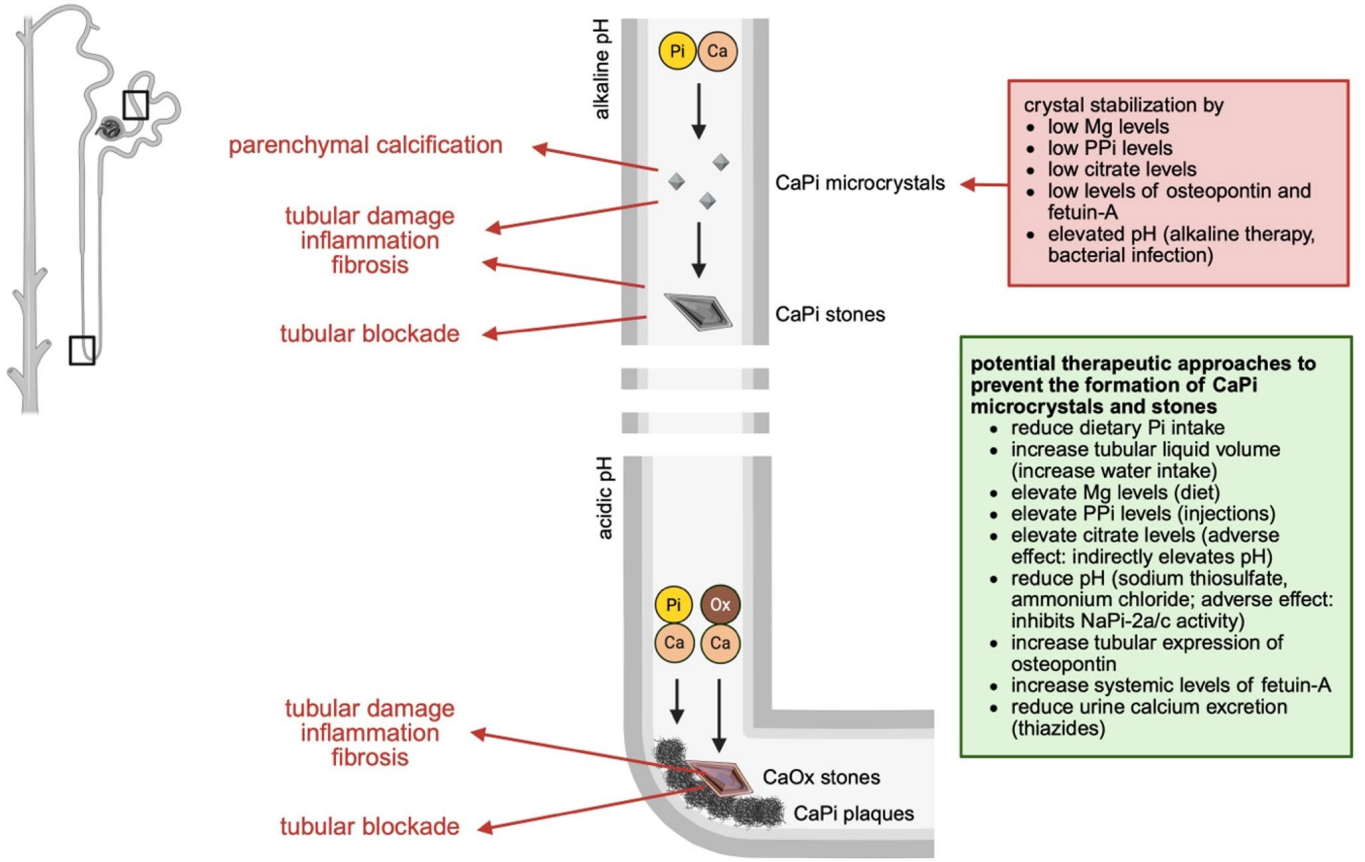
The contribution of hyperphosphaturia to the formation of microcrystals and kidney stones and to kidney injury Increases in tubular concentrations of phosphate (Pi) and calcium (Ca) in the proximal tubule can result in the formation of calcium phosphate (CaPi) microcrystals which can precipitate in the tissue and cause damage. CaPi microcrystals can also induce kidney damage by activating specific signaling events on tubular epithelial cells. Microcrystals can also grow to form CaPi stones which also damage the kidney. Further downstream in the thin loops of Henle, Ca and Pi form plaques (so called Randall’s plaques), which serve as nucleation sites for the formation of calcium oxalate (CaOx) stones. Mg: magnesium; PPi: pyrophosphate

Phosphate infusion and micropuncture studies in rats have shown that the phosphate concentration in the tubular fluid increases along the proximal tubule and that in CKD tubular phosphate levels might be sufficiently high to result in the formation of phosphate particles, such as calcium phosphate microcrystals [22]. Indeed, several of the CKD animal models with hyperphosphaturia develop nephrocalcinosis as part of their renal pathology [56, 58, 60]. In nephrectomized rats receiving a high phosphate diet the degree of renal calcification correlates with the overall histological damage [51]. Moreover, studies in nephrectomized rats on a high phosphate diet have shown that inhibiting calcification by administration of diphosphonate or 3-phosphocitric acid reduces renal injury and renal dysfunction [51, 54, 61]. These studies suggest that the deposition of calcium phosphate microcrystals in the tubular lumen contributes to progressive kidney injury in CKD (Fig. 3) [70]. This concept is further supported by human CKD studies with the histological detection of calcium phosphate microcrystals in kidney biopsies and autopsies [71, 72]. A study in ESRD patients has shown that calcium phosphate microcrystals cannot only be detected in the tubular lumen, but also within the tissue, and are mostly present in the extracellular matrix of tubular epithelial cells in different nephron segments [73]. These tissue crystals seem to appear before calcifications and can be detected by standard histological stains and microscopy, suggesting that microcrystals might serve as precursors of calcifications. Overall, it appears that the kidney accumulates calcium phosphate microcrystals early during the course of CKD prior to serum phosphate elevations.

In vitro studies in proximal tubular epithelial cells have shown that phosphate elevations in culture medium cause the formation of calcium phosphate microcrystals, which is accompanied by the osteogenic transdifferentiation and calcification of cells [74]. A mechanistic study found that that prolonged exposure of tubular epithelial cells to calcium phosphate microcrystals leads to the endocytosis of these microcrystals via toll-like receptor 4 (TLR4) [61]. The calcium phosphate microcrystals also activate pro-inflammatory signaling pathways independently of TLR4. The same study found that mice exposed to high phosphate diets have luminal depositions of calcium phosphate crystals in the proximal tubule which is associated with increased expression of markers of tubular injury, inflammation, and fibrosis [61]. These pathologic effects are dependent on the expression of TLR4, as TLR4-deficient mice show no signs of tubular crystal deposition. Overall, it appears that calcium phosphate microcrystals accumulate in proximal tubular epithelial cells, disturb endosomal trafficking, and cause cell damage, inflammation, and tubulointerstitial fibrosis, and thereby contribute to the progression of CKD. This experimental study shows that an increase in the phosphate concentration in the proximal tubular fluid accelerates nephron loss in CKD and supports a direct role of microcrystal formation as a driver of tubular damage in CKD [75]. However, whether the high concentrations of calcium and phosphate used in cell culture experiments can be reached in the proximal tubular lumen of animals and humans and whether this pathomechanism exists in patients with CKD remains to be determined.

FGF23 and PTH inhibit phosphate reabsorption by renal tubular epithelial cells and thereby increase phosphate excretion per nephron and phosphate concentrations in the proximal tubular fluid (Fig. 2). The above mechanism would suggest that while systemic elevations of FGF23 and PTH protect from the pathological actions of serum phosphate increases, at the same time they promote the formation of calcium phosphate microcrystals by increasing phosphate concentrations in the proximal tubule and thereby contribute to renal injury. In fact, continuous injections of PTH in healthy rats serve as an animal model for nephrocalcinosis [76]. Patients with CKD might be particularly at risk because of the combination of reduced nephron number and increased production of FGF23 and PTH [61]. This is supported by a recent cross-sectional study in pre-dialysis patients reporting that increases in tubular phosphate levels are associated with elevations in serum levels of FGF23 and in markers of tubular damage [66]. Overall, it appears that elevated FGF23 does not directly damage kidney cells [77], but contributes to the progression of kidney injury by elevating tubular phosphate levels and causing calcium phosphate crystal deposits.

The kidney is not defenseless against these pathologic alterations and has developed mechanisms to protect itself from the formation of calcium phosphate crystals and their harmful actions on the tubular epithelium. Osteopontin is a cytokine with various functions that is produced by different tissues. By preventing the growth and maturation of calcium phosphate crystals, osteopontin acts as a local calcium phosphate buffer to inhibit mineral deposition and to regress calcification [78]. Phosphate is an inducer of osteopontin expression in various cell types [79, 80], and calcium phosphate microcrystals increase osteopontin expression in renal tubular cell lines [50, 61]. Renal osteopontin levels are elevated in animal models of CKD and hyperphosphaturia [81, 82], and osteopontin serves as a marker for renal tubular damage [83]. Mice lacking osteopontin show more severe renal calcifications following the induction of CKD or the administration of a high phosphate diet, suggesting that osteopontin inhibits nephrocalcinosis and that this mechanism might fail in CKD [50, 82]. Similarly, fetuin-A is a liver-derived calcium phosphate buffer that acts as a systemic inhibitor of calcifications [84, 85]. Serum fetuin-A levels are reduced in CKD [86–88], and the induction of CKD combined with the administration of a high phosphate diet causes renal calcifications in fetuin-A knockout mice [89]. Although fetuin-A is not expressed in the kidney, it can pass the glomerular filter and enter the tubular lumen, where it might block the formation of calcium phosphate microcrystals [76]. Furthermore, since the overall NaPi cotransporter activity in the proximal tubule is modulated by extracellular pH, changes in the pH might affect nephrocalcinosis. An alkaline pH increases phosphate uptake rates [90], whereas acidosis reduces NaPi-2a/c activity and cell surface expression [8, 91, 92], suggesting that at an acidic pH, phosphate concentrations in the tubular lumen should be higher. Since a low pH protects from the formation of calcium phosphate crystals, an acidic pH might elevate tubular phosphate concentrations while at the same time preventing calcium phosphate crystal formation (Fig. 3). It is possible that this physiologic mechanism exists so that renal phosphate excretion increases in acidosis to remove excess protons as titratable acid [92].

Overall, it appears that the harmful effects of hyperphosphaturia are mediated by the direct actions of calcium phosphate microcrystals on tubular epithelial cells, which results in tubular cell damage and parenchymal calcifications. Furthermore, nephrocalcinosis is accompanied by renal fibrosis and inflammation [19]. While tubular injury can promote tubulointerstitial fibrosis and inflammation, microcalcifications themselves may directly activate fibroblasts and inflammatory cells in the kidney and thereby directly contribute to renal injury that goes beyond calcifications, as shown in other tissues [16]. Nephrocalcinosis is usually defined by the deposition of calcium phosphate microcrystals in the renal tubules and parenchyma and not in the renal vasculature [18]. However, calcifications of the renal arteries and their branches have been reported in patients with type-2 diabetes where they seem to associate with the rapid progression of kidney disease and mortality [93, 94]. Since by targeting vascular smooth muscle cells phosphate is a potent inducer of calcification in various vascular beds [17], it would be expected that renal arteries, arterioles and veins also calcify in the context of hyperphosphatemia. While most experimental and clinical studies on nephrocalcinosis focus on tubular and parenchymal pathologies, it would be important to precisely localize calcifications, and distinguish between vascular and non-vascular calcifications within the kidney, which most likely will differ in their functional consequence for the kidney. Furthermore, it would be important to identify cell types that contribute to non-vascular calcifications in the kidney. It has been shown in a rat model of hyperglycemia that podocytes in the glomerulus can calcify, and in vitro studies suggest that podocyte calcification is induced by high glucose levels [48]. Moreover, a transgenic rat model with increased phosphate uptake by podocytes develops glomerular injury that progresses to kidney damage [95]. Direct pathologic effects of phosphate on the glomerular filter have been shown in rats with repetitive i.v. injections of phosphate, which causes calcifications in different glomerular areas, including the glomerular basement membrane, as well as podocyte damage and proteinuria [96]. Calcium phosphate depositions in the glomerular basement membrane have been also detected in the adenine mouse model of CKD [60]. However, the pathological relevance of phosphate effects on the glomerulus in CKD patients remains to be established. Furthermore, it would be important to determine whether elevated phosphate can contribute to primary podocyte injury with proteinuria, as in patients with nephrotic syndrome, before they progress to CKD. To date, only a few clinical studies have analyzed phosphate metabolism in the context of nephrotic syndrome, and findings on serum levels of phosphate and FGF23 have been inconsistent [97–101]. The same is the case for studies in animal models of nephrotic syndrome [102–108]. It would be important to expand on these pre-clinical and clinical studies and approach nephrotic syndrome in the context of potential changes in mineral metabolism.

While intrarenal parenchymal calcifications seem to be driven by tubular calcium phosphate microcrystals, it cannot be excluded that other circulating factors coming from the blood promote the calcification process, which need to be identified. Furthermore, the formation of calcium phosphate microcrystals in the blood is prevented by fetuin A, which acts as a sponge that binds calcium and phosphate [84, 85]. The most primitive form of these particles are calciprotein monomers (CPM) with a single fetuin-A molecule bound to calcium phosphate clusters. CPMs can cumulate to form calciprotein particles (CPPs), which have a larger size and higher density and can transition from an amorphous to a crystalline phase [109]. Amorphous CPMs and CPPs are dispersed in solution and can be filtered and removed by the kidney via megalin-mediated endocytosis in the proximal tubule or by renal excretion [110, 111]. In contrast, solid crystalline CPPs cannot pass the glomerular filter and are cleared from the circulation by sinusoidal endothelial cells and Kupffer cells in the liver and by macrophages in the spleen [109, 111–114]. Interestingly, CPPs have been detected in the blood of CKD patients, even in early disease stages when serum phosphate levels are not significantly elevated ted [115, 116]. Vascular calcifications also occur in early stages of CKD prior to the development of hyperphosphatemia [117], and serum CPP levels positively correlate with vascular calcification in CKD patients [115, 116, 118]. These clinical findings suggest that CPPs might contribute vascular calcification, as supported by in vitro studies in vascular smooth muscle cells [119, 120]. Interestingly, elevations in serum concentrations of CPMs and CPPs predict CKD progression [121, 122]. Whether elevated levels of amorphous CPMs and CPPs in the tubules or of crystalline CPPs in the circulation can cause parenchymal or vascular calcifications and renal damage needs to be determined. Furthermore, CPPs contain various other biomolecules, including proteins, which form a corona around the microcrystals, and many of the known CPPs’ biological and pathological effects are caused by the corona and not the inorganic crystals [111]. In general, in biological fluids “naked” inorganic crystals do not exist but are almost always coated with proteins [111]. It will be important to determine if microcrystals formed within the tubular lumen also contain a biomolecule corona, which might mediate the observed pathologic actions of the renal calcium phosphate microcrystals.

## Hyperphosphaturia contributes to the formation of kidney stones

In contrast to nephrocalcinosis with diffuse renal parenchymal calcifications, nephrolithiasis is the formation of kidney stones, which are larger mineral deposits in the renal collecting system [20]. Kidney stone disease affects about 10% of the Western population, with recurrence in about 50% of patients, thereby generating a large clinical problem [123]. Obesity, diabetes and hypertension are major risk factors for stone formation, and over the past 50 years the prevalence of kidney stones has been consistently growing and is expected to further increase based on changes in lifestyle and dietary habits [20]. Kidney stones can form when minerals in the urine are supersaturated, leading to crystal formation, growth, aggregation and retention within the kidney. Stones contain crystalline and organic components and are found free or attached to the renal papillae. About 85% of kidney stones contain calcium, and about 5% of these stones consist of pure calcium phosphate crystals, which in the urine sediment are typically dark and amorphous [20, 124, 125]. Calcium phosphate stones can be composed of different forms, with hydroxyapatite being the most common one [126]. Tricalcium phosphate, also called Whitlockite, and calcium monohydrogen phosphate, also called Brushite, are less common, but are associated with more severe kidney damage and higher risk of recurrent stone formation. An acidic pH and citrate are known inhibitors of calcium phosphate crystal formation, and Brushite is usually observed in the context of high urine pH and low urine citrate concentrations [127]. Carbonate apatite and magnesium ammonium phosphate, also called struvite, are other forms of calcium phosphate stones, which mainly occur when bacteria produce ammonia and the urine pH is elevated [128]. These types of stones can form following urinary tract infections and are also referred to as “infection stones”.

While pure calcium phosphate stones are rare [125], the majority of kidney stones consist of calcium oxalate, with about 80% of them also containing calcium phosphate, although usually in small quantities [20, 124]. Randall’s plaques are microscopic calcium phosphate deposits in the basement membranes of the thin loops of Henle [129], which act as anchored nucleation sites for the formation of calcium oxalate crystals [130–132], explaining the co-existence of calcium oxalate and calcium phosphate in the majority of kidney stones. While several mechanisms of stone formation have been proposed [20], it is possible that calcium phosphate microcrystals might serve as a major inducer of the process (Fig. 3). It is well established for vascular calcification that calcium phosphate microcrystals cause the osteogenic differentiation of vascular smooth muscle cells thereby driving the calcification process [17]. It is possible that calcium phosphate microcrystals also induce the de-differentiation and potentially osteogenic differentiation of renal tubular epithelial cells, which then promotes the deposition of calcium phosphate crystals and the formation of Randall’s plaques [20]. Therefore, it is plausible to assume that increases in tubular concentrations of phosphate contribute to kidney stone formation and growth. This is supported by rare genetic diseases, where carriers of genetic loss-of-function variants of NaPi-2a and NaPi-2c have hyperphosphaturia and a higher risk of developing kidney stones [133, 134]. Moreover, mice deficient in NaPi-2a are phosphaturic and calciuric and form renal calcium oxalate crystal deposits when treated with either hydroxyproline or glyoxylate as metabolic precursors of oxalate, which does not occur in wildtype mice [58, 135]. However, whether increases in tubular concentrations of phosphate contribute to the formation of Randall’s plaques is currently not known. A microscopic analysis of the kidneys from NaPi-2a knockout mice with hyperphosphaturia suggests that the formation of calcium phosphate microcrystals occurs in the tubular lumen [136]. These tubular microcrystals then seem to act as nuclei that grow by the addition of more microcrystals to the periphery, leading eventually to the formation of crystals large enough to occlude the tubular lumen. Interestingly, NaPi-2a knockout mice do not show calcium phosphate deposits in the basement membrane of the loops of Henle [136], where Randalls’ plaques in human kidneys form. This animal study questions whether Randall’s plaques are actually needed to promote the formation of calcium phosphate stones.

While lifestyle, diet, and water intake are major contributors to the development of kidney stones, there is growing evidence for a critical role of inherited susceptibility in nephrolithiasis [137, 138], which is estimated to affect about a third of the patients [139, 140]. A subset of these patients have a Mendelian monogenic stone disease, with approximately 40 genes currently identified causing childhood- and adult-onset disease [141, 142]. In these patients nephrocalcinosis is commonly observed and associated with a greater risk of CKD [143]. Interestingly, recent genome-wide association studies (GWAS) and population exome studies identified genetic variants of NaPi-2a and NaPi-2c and of other regulators of mineral metabolism that increase the risk for kidney stones [144–149]. In vitro studies suggest that some of the identified NaPi-2a variants have reduced transporter activity or expression levels [150, 151]. Many genetic forms of nephrolithiasis seem not to be caused by monogenic but polygenic inheritance [152], suggesting a more complex pathomechanism. This is supported by the identification of heterozygous rare variants in NaPi-2c in larger populations of unrelated kidney stone formers [153]. Overall, these human genetic studies suggest that hyperphosphaturia contributes to nephrolithiasis. If true, reduced nephron numbers, as in CKD, or high dietary phosphate intake should also increase the risk of kidney stone formation. However, while many patients with calcium phosphate stones have high tubular levels of calcium (hypercalcuria) [127], studies have been inconsistent in their findings on urinary phosphate concentrations in stone formers compared to healthy individuals. Moreover, whether the phosphaturic action of FGF23 and PTH, which elevates tubular phosphate concentrations, contributes to stone formation is also unknown. However, patients with primary hyperparathyroidism are at risk for developing kidney stones [154], with the occasional observation of pure calcium phosphate stones [155]. Furthermore, elevated FGF23 and reductions in klotho levels have been reported in kidney stone formers [156–163]. Therefore, by reducing phosphate uptake in the proximal tubules and increasing tubular phosphate levels, elevated FGF23 might promote stone formation. However, FGF23 has been also shown to induce calcium uptake by the distal renal tubules via FGFR/klotho signaling and to increase membrane abundance of the calcium channel transient receptor potential vanilloid-5 (TRPV5) [164]. This physiological effect of FGF23 should lower tubular calcium levels thereby reducing the risk of stone formation. Thus, the potential relationship between changes in FGF23 levels and nephrolithiasis requires further investigations.

Although calcium oxalate and calcium phosphate coexist in most kidney stones, there are significant differences among both types of crystals [20]. For example, while magnesium and citrate block the formation of calcium oxalate and calcium phosphate crystals [165, 166], the pH has opposite effects on the two types of crystals. An acidic pH inhibits the formation of calcium phosphate crystals but promotes the formation of calcium oxalate crystals [167], and therefore a higher urine pH distinguishes calcium phosphate stone formers from calcium oxalate stone formers. Calcium phosphate crystals form in the earlier segment of the nephron where the urine is alkaline, whereas calcium oxalate crystals form in distal tubules and collecting ducts where the urine is slightly acidic (Fig. 3) [130, 131]. It seems that pure calcium phosphate stones can grow faster and larger than calcium oxalate stones [20]. Moreover, although males are two times more likely to develop kidney stones in general, females are more likely to have calcium phosphate stones [127, 168]. The underlying pathomechanisms for these observations are not clear. Based on these differences, it is likely that one therapeutic approach might not be effective to tackle all types of kidney stones [127]. Furthermore, alkali therapy commonly used to treat metabolic acidosis in CKD might prevent calcium oxalate stone formation but may also promote calcium phosphate stone formation [169].

Stone formers have an increased risk for developing CKD and ESRD [20]. Experimental studies have shown that calcium oxalate crystals [170–176] and calcium phosphate crystals [177] can injure renal epithelial cells and alter the response and metabolism of immune cells, suggesting that kidney stones can directly contribute to kidney damage [178, 179]. If kidney stones cause CKD, they should also induce hyperphosphatemia, as shown in a mouse model receiving an oxalate-rich diet [180]. By doing so, kidney stones would increase tubular phosphate concentrations and further promote the formation and growth of calcium phosphate stones. Therefore, the formation of kidney stones and the concurrent kidney damage could be the result of the same pathological stimulus, which may include hyperphosphaturia. Overall, while hypercalcuria represents the most important metabolic feature of kidney stone disease, there is a lack of awareness regarding phosphate abnormalities in this disease [181]. Future experimental and clinical studies should focus on the potential impact of phosphate on kidney stone formation.

## Dietary phosphate restriction reduces kidney injury progression in CKD

Studies lowering dietary phosphate content in animal models of CKD have demonstrated that hyperphosphatemia directly contributes to kidney injury, and suggested that dietary phosphate restrictions may exert reno-protective effects in patients with CKD (Fig. 2). For example, it has been shown that the administration of a lowphosphate diet to rat models of CKD with subtotal nephrectomy reduces calcium phosphate content and alleviates injury of the remnant kidney, including interstitial fibrosis and inflammation, thereby stabilizing residual kidney function and extending the animals’ lifespan [44–46, 52, 55, 182–185]. Similarly, diet supplementation with phosphate binders to deplete phosphate or the blockade of gastrointestinal phosphate uptake in nephrectomized rats protects the kidney and improves kidney function [46, 186–189]. Furthermore, administration of a low-phosphate diet to mice with global deletion of collagen 4a3 (*Col4a3*^*−/−*^), which is a model of human Alport Syndrome with glomerular damage that progresses to CKD, ameliorates renal tubular injury and inflammation, without affecting the glomerular pathology [49]. Reno-protective effects of low-phosphate diets and the blockade of gastrointestinal phosphate uptake have also been described in other animal models of glomerular disease, such as glomerulonephritis and IgA nephropathy [190–192]. Furthermore, dietary phosphate restriction reduces kidney cyst burden and interstitial fibrosis in a genetic mouse model of PKD [193]. Similarly, it was found that a reduction in phosphate consumption in dogs and cats with established kidney disease reduced renal calcification and injury, slowed kidney disease progression, and prolonged survival [41, 42, 194]. Cats have a high incidence of kidney disease [195–197], in part due to the development of nephrocalcinosis with aging [198], and it has been shown that the administration of a low phosphate diet reduced the degree of kidney calcification in growing kittens [199]. Several of these animal studies have shown that the administration of a phosphate-restricted diet slows down the progression of kidney injury when compared to animals fed phosphate-repleted diets, despite similar intake of protein and other nutrients [42, 44, 52, 186, 190, 191]. These studies suggest that dietary phosphate restrictions preserve kidney function which occurs independently of the reno-protective effects of reduced dietary protein [184]. However, it is important to note that not all experimental studies that lowered serum phosphate levels in animal models of CKD detected beneficial effects on the kidney. For example, the global deletion of NaPi-2b in mice with CKD, induced by an adenine-rich diet or subtotal nephrectomy, reduced serum phosphate levels but did not result in an improvement in kidney function [200].

Since serum phosphate levels rise late in the progression of CKD and may contribute to cardiovascular disease, most clinical studies and trials with dietary phosphate restrictions and phosphate binders have been conducted in ESRD patients with a focus on lowering serum phosphate and FGF23 to reduce cardiovascular events and mortality [201–204]. However, small clinical studies suggest that low phosphate diets slow the progression of kidney injury in pre-dialysis CKD patients [205–209], supporting the concept that dietary phosphate consumption is a modifiable risk factor for CKD progression in humans and the importance of phosphate restriction for kidney health. However, not all clinical studies found a benefit of dietary phosphate restriction on CKD progression [210]. Furthermore, clinical trials studying phosphate binders in pre-dialysis CKD patients did not provide evidence for potential kidney-protective effects [211–213]. However, it is important to note that these studies were not specifically designed to examine kidney function as the primary outcome, and more importantly, they could not demonstrate a clear effect on lowering serum phosphate levels or urinary phosphate excretion. Randomized controlled trials in pre-dialysis CKD patients are needed to test whether dietary phosphate restriction attenuates kidney disease, as supported by feeding studies in animal models of CKD discussed above. Furthermore, based on the potential contribution of hyperphosphaturia to kidney stone formation, it would be worth investigating whether reduced dietary phosphate intake lowers the risk of kidney stone formation in humans.

## Effects of phosphate elevations on the healthy kidneys

High dietary phosphate intake in healthy individuals for only a few days can increase the phosphate levels in serum and urine [21]. Moreover, elevated serum phosphate concentrations are associated with high mortality in individuals with preserved kidney function [214–216]. Epidemiological studies have shown that individuals with higher phosphate levels are more likely to develop CKD [217, 218]. Combined, these observations suggest that high dietary phosphate intake can cause hyperphosphatemia and hyperphosphaturia in individuals with normal kidney function, and that increases in dietary phosphate might be a significant health risk for healthy individuals, which includes the induction of kidney injury (Fig. 2). This hypothesis is supported by experimental studies showing that the administration of high phosphate diets to healthy rats or mice induces kidney damage, including proximal tubular injury and tubulointerstitial fibrosis and inflammation [51, 53, 67, 219–221]. Signs of proximal tubular injury appeared within days, which then worsened within a few weeks [222–225]. Feeding with polyphosphates, which are linear chains of phosphates, caused more severe renal tubular damage and fibrosis than feeding of monophosphate [221, 223, 224]. Similarly, the administration of a high phosphate diet reduced kidney function in healthy cats within weeks [226, 227]. Many of these long-term feeding studies in animals report the development of hyperphosphatemia and hyperphosphaturia [53, 67, 220, 221, 228]. Therefore, the mechanism of kidney injury following high phosphate intake could be similar to the one discussed earlier for CKD, which is driven by increased tubular phosphate concentrations with a contribution of FGF23 and PTH. Indeed, studies in healthy rats have shown that the administration of a high phosphate diet causes proximal tubular cell injury, including the accumulation of calcium phosphate microcrystals in lysosomes and mitochondria [222]. Calcium phosphate microcrystals appeared already after a few days following the introduction of a high phosphate diet, and crystals further increased in size within weeks [229]. Tubular areas with calcium phosphate accumulation expressed osteopontin [225], suggesting the activation of a similar reno-protective mechanism as proposed for CKD. In general, the kidney pathology in rodents receiving high phosphate diets includes nephrocalcinosis where calcium phosphate microcrystals appear in the renal tubules and in the parenchyma [51, 53, 219–225, 230–234].

Acute phosphate elevations also seem to damage the kidney. For example, daily phosphate injections in rats induce renal calcification within 6 to 14 days [96, 235, 236]. Similarly, human studies have shown that the acute intake of high phosphate can damage the kidney in the absence of pre-existing kidney disease [237, 238]. For example, patients preparing for coloscopy or colonic surgery by ingesting oral sodium phosphate solutions as a laxative to clean the digestive tract can develop nephrocalcinosis [239]. These phosphate solutions induce transient hyperphosphatemia [240, 241], which can reach extremely high levels and result in acute kidney injury (AKI) and renal failure [242–254]. The acute phosphate nephropathy is characterized by phosphate deposits in the renal tubular lumina and epithelia [239, 249], suggesting that increases in tubular phosphate levels might cause the observed kidney damage. Furthermore, the rapid release of phosphate from internal stores might contribute to AKI that occurs in the context of rhabdomyolysis and tumor lysis syndrome [238]. In general, increased serum phosphate levels have been associated with a higher risk of developing AKI in hospitalized patients [255]. As discussed for CKD, phosphate elevations might not necessarily cause kidney damage but contribute to the progression of kidney injury following the induction of AKI by other pathologic stimuli. In fact, hyperphosphatemia seems to be common in AKI, including patients without acute primary phosphate elevations [238]. Furthermore, animal models of AKI show rapid increases in serum phosphate levels upon injury induction by folic acid injections or renal ischemia-reperfusion [256–259]. One would assume that these secondary elevations in serum phosphate levels following the induction of AKI are mainly caused by decreased renal phosphate excretion due to the kidney injury [238]. However, a recent study in mice with folic acid-induced AKI showed that hyperphosphatemia was not accompanied by changes in urinary phosphate excretion [256]. It is possible that renal phosphate uptake is elevated in AKI, as suggested by the increased renal expression of the phosphate transporters NaPi-2b and PiT-1 in mice with AKI induced by renal ischemia-reperfusion [259]. Clearly, the mechanisms driving hyperphosphatemia in AKI need further investigation. Furthermore, since AKI does not seem to be accompanied by hyperphosphaturia, pathologic actions of phosphate on the kidney might be based on hyperphosphatemia. A recent study in mice with folic acid-induced AKI showed that a low phosphate diet reduced serum phosphate levels and ameliorated kidney injury [256]. Furthermore, increased serum phosphate levels have been associated with a higher risk of short-term mortality among patients with established AKI [260], and a low-phosphate diet in AKI mice also had beneficial effects on the heart and increased survival [256]. It is possible that the pathologic actions of phosphate elevations in AKI are mediated by increases in serum CPP levels [256] and by the associated elevations in systemic levels of FGF23 and PTH and decreases in 1,25D and klotho [238]. Although the precise underlying pathomechanisms need further investigation, it appears that reducing dietary phosphate intake might be an approach to tackle AKI and associated pathologies, which should be studied in AKI patients.

Since renal excretion is quickly adapted to acute and chronic phosphate intake, it is unclear why phosphate feeding or injection studies in the absence of kidney damage cause hyperphosphatemia despite elevations in FGF23 and PTH and increased renal phosphate excretion [64]. Furthermore, without reductions in nephron numbers it is unclear how increased phosphate intake can increase tubular phosphate to levels that result in the formation of calcium phosphate microcrystals and nephrocalcinosis. In fact, not all phosphate feeding studies are consistent in their findings, as some phosphate loading studies in rodents with normal kidney function did not elevate serum phosphate levels [261–263]. Furthermore, some animal studies have shown that a high-phosphate diet by itself does not damage the kidney. For example, administration of a high-phosphate diet in mice for one year induced hyperphosphaturia and hyperphosphatemia and caused bone damage, but had no effect on kidney function [264]. Moreover, a high phosphate diet in mice for 12 to 16 weeks induced hyperphosphatemia but no signs of kidney injury [60, 265, 266]. In a recent study, the administration of a high phosphate diet in mice for 14 weeks resulted in increased urinary phosphate excretion without changes in serum phosphate levels or signs of kidney injury [267]. The discrepancy in findings from different feeding studies might be based on the different rodent models. For example, it has been shown in rats that the effect of a high phosphate diet on inducing nephrocalcinosis is dependent on the rat strain that was studied [268]. Furthermore, in mice the development of nephrocalcinosis seems to be age-dependent, and only occurs when the high phosphate diet was introduced in weaned but not in periadolescent mice [230]. Furthermore, the effects of a high phosphate diet on the kidney might be sex-dependent, and nephrocalcinosis seems to be more severe in female than in male rats [269]. Differences between studies could also be based on differences in the dietary sources of phosphate which might differ in their effect on phosphate homeostasis [270–272], or on the circadian rhythm in the regulation of serum phosphate levels and differences in the time of day when samples were collected [6]. Ultra-processed foods have a high content of phosphate salts that are fully absorbed in the small intestine. Since a significant portion of the population is consuming twice the amount of the recommended amount of phosphate on a daily base [64], it will be important to determine whether a chronic consumption of a high phosphate diet in the context of normal kidney function can lead to hyperphosphaturia and to sustained increases in serum phosphate levels, which could cause kidney damage as well as widespread ectopic calcifications.

CKD of unknown etiology (CKDu) is a mysterious tubulointerstitial renal disease that is unrelated to the traditional risk factors of CKD [273]. CKDu occurs in low-income tropical countries within farming communities and is associated with high mortality rates [274–276]. It appears that the affected individuals live in dry zones at higher elevations, and consume hard water, which is rich in calcium carbonate [277]. Their drinking water is also rich in phosphate based on the overuse of phosphate containing fertilizers for agriculture in these distinct areas [278]. Moreover, the natural weathering process constantly releases fluoride into the groundwater [279]. Fluoride can integrate into calcium phosphate apatite thereby creating fluorapatite crystals with even higher stability, which is the base for the formation of dental enamel. The intake of groundwater concentrated with calcium, phosphate and fluoride, combined with chronic dehydration, seems to provide a perfect condition for the formation of stable crystals in the renal tubular system [277]. However, whether such crystals really exist in the kidney is currently unknown. Interestingly, individuals living in areas with higher natural fluoride levels in the groundwater seem to have higher urinary fluoride excretion, higher urinary levels of osteopontin, and a higher incidence of kidney stones [280, 281]. In vitro studies suggest that fluoride promotes stone formation [282], and fluoride can be detected in kidney stones [283]. However, a study in a rat model with induced kidney stone formation reported that the administration of fluoride with the drinking water protected from kidney stones, which seemed to be based on a reduction of oxalate synthesis [284]. Clearly, more research is needed to determine the potential pathologic effects of fluoride on the kidney and whether they occur in combination with hyperphosphaturia. If true, providing fresh drinking water with lower calcium carbonate, phosphate and fluoride content and increasing water consumption might prevent the development of CKDu.

Genetic diseases with reductions in renal phosphate uptake further support the hypothesis that chronic phosphaturia can cause kidney damage (Fig. 2) [3]. For example, genetic loss-of-function variants of NaPi-2a and NaPi-2c have been reported in patients with Idiopathic Infantile Hypercalcemia (IIH) [285–287] and with Hereditary Hypophosphatemic Rickets and Hypercalcuria (HHRH) [288, 289], who both develop hyperphosphaturia, resulting in renal phosphate wasting, hypophosphatemia and the development of rickets. Many carriers of these genetic variants develop nephrocalcinosis or kidney stones, and they have an increased risk of developing CKD in adulthood [133, 134, 287, 290–292]. Furthermore, a study in patients with HHRH reported an association with kidney cysts [293]. In general, NaPi-2a and regulators of NaPi-2a expression have been frequently identified as gene loci that associate with an increased risk of CKD and a decline in kidney function [294–298]. Overall, these findings in humans are in line with experimental studies showing that NaPi-2a knockout mice develop hyperphosphaturia and calcium phosphate deposits in the renal tubules [81, 135, 136, 299]. Moreover, patients with X-linked hypophosphatemia (XLH) carry mutations in *Fgf23* or in genes encoding for regulators of FGF23 expression, which results in uncontrolled FGF23 elevations, hyperphosphaturia and rickets. Some XLH patients develop nephrocalcinosis and nephrolithiasis as well as reduced kidney function [300, 301]. A recent case report with a detailed analysis of kidney biopsies detected proteinuria and glomerular damage in a patient with XLH [302]. Furthermore, this study showed dilations in the proximal tubules as well as reduced expression of NaPi-2a and NaPi-2c and the appearance of lysosomal particles in proximal tubular epithelial cells. Overall, more clinical studies are needed to determine whether XLH is associated with an increased risk for developing CKD, which will not be an easy task based on challenges in estimating kidney function in this patient population [303].

Genetic diseases with hyperphosphatemia, which secondarily can lead to hyperphosphaturia to reduce serum phosphate levels, provide further evidence that phosphate elevations can damage the kidney (Fig. 2). For example, impairments in the phosphaturic FGF23-klotho system lead to hyperphosphatemia. Mutations in *Klotho* and in *Fgf23* as well as in genes encoding for the regulators of FGF23 production cause familial tumoral calcinosis [304–307], which is a rare genetic disease with hyperphosphatemia and ectopic calcifications [308], including nephrocalcinosis with decreased renal function [309, 310]. Furthermore, genetic mouse models with deficiencies of *klotho* (*kl/kl*) or *Fgf23* develop hyperphosphatemia and ectopic calcifications, including renal calcifications as well as other renal pathologies, such as fibrosis and cell apoptosis and senescence [57, 77, 311–328]. Deletion of NaPi-2a in *kl/kl* mice, which lowers serum phosphate levels, has reno-protective effects while the administration of a high phosphate diet to these mice reintroduces renal injury [311, 322]. Furthermore, a low phosphate diet reduces renal calcification in *kl/kl* mice [312]. Combined, these studies in human genetic diseases and in their corresponding animal models suggest that in the absence of pre-existing kidney injury hyperphosphaturia can induce kidney damage.

While most disorders of renal phosphate wasting are of genetic origin [329], phosphaturia can also be an acquired condition, for example induced by drugs [330]. Acquired phosphaturia causes hypophosphatemia, which can result in rickets in children and osteomalacia in adults [330]. Furthermore, acquired phosphaturia can lead to progressive kidney injury and potentially renal failure [330]. For example, FDA-approved inhibitors against FGFRs, such as Pemigatinib, Erdafitinib, and Infigratinib, are used as chemotherapies against some types of tumors with gain-of-function mutations or increased activity of FGFRs, including cancer of the bladder and bile duct. FGF23 mediates its physiological effect in the kidney via FGFRs, and hyperphosphatemia is common in cancer patients receiving FGFR inhibitors, occurring in 60–85% of clinical trial patients [331]. Hyperphosphatemic tumoral calcinosis has been described in some of the cancer patients receiving FGFR inhibitors [332], which seemed to improve when the patients received oral phosphate binders [333]. Whether these cancer patients also develop nephrocalcinosis is not known. However, healthy rats receiving a pan-FGFR inhibitor develop hyperphosphatemia and widespread ectopic calcifications, including renal calcifications [334].

Studying human diseases and animal models with a loss of mechanisms that protect from calcium phosphate crystal formation can also provide evidence that phosphate can damage the kidney. A multi-layered safety net of physiologic and cellular processes ensures that elevations in phosphate levels do not result in the uncontrolled formation of calcium phosphate crystals outside of the bone [16]. As discussed earlier, fetuin-A and osteopontin serve as inhibitors for the formation of calcium phosphate crystals, and the elevation of phosphate levels, for example by administration of a high phosphate diet, in genetic mouse models lacking fetuin-A [335–337] or osteopontin [50, 82] induces ectopic calcifications, including nephrocalcinosis. Pyrophosphate (PPi) is a metabolite that is generated by many cells and that blocks the formation of calcium phosphate crystal [338]. Extracellular ATP that is released from the liver via ATP Binding Cassette Subfamily C Member 6 (ABCC6) serves as the major source of systemic PPi [339]. Ectonucleotide pyrophosphatase/phosphodiesterase 1 (ENPP1) is a transmembrane protein expressed by various cell types that rapidly hydrolyzes extracellular ATP into AMP and PPi, thereby acting as the primary producer of extracellular PPi in the body [340]. Genetic mouse models with deficiencies in *Abcc6* [341–345] or *Enpp1* [346–348] have reduced PPi levels and develop ectopic calcifications, including nephrocalcinosis. The pathology seems to worsen when mice are administered a high phosphate diet [346]. Patients with *Enpp1* mutations develop generalized arterial calcification of infancy (GACI) or autosomal recessive hypophosphatemic rickets type 2 (ARHR2) [340]. While oral phosphate supplementation improves the skeletal symptoms in these patients, it seems to worsen ectopic calcifications, including renal calcification [349]. Pseudoxanthoma elasticum (PXE) is an autosomal recessive disease with *Abcc6* deficiency resulting in reduced serum PPi levels and ectopic calcifications [350], including nephrocalcinosis and nephrolithiasis and the formation of Randall’s plaques [351–356]. Tissue-nonspecific alkaline phosphatase (TNAP) is a ubiquitously expressed enzyme that is anchored on the outer side of the cell membrane and that hydrolyzes PPi, thereby reducing local PPi and elevating phosphate levels [357]. Transgenic mice which overexpress TNAP develop ectopic calcifications, including renal calcifications [358]. Similar to PPi, magnesium blocks the calcium phosphate crystal formation [359]. Magnesium is an essential mineral, and systemic magnesium levels are regulated by intestinal absorption, bone exchange and renal excretion [360]. Administration of PPi or magnesium to mice with fetuin-A deficiency prevents renal calcifications [337]. In contrast, lowering magnesium levels in mice with genetic deficiency for *Enpp1* worsens calcifications [346]. Overall, the loss of mechanisms that protect from calcium phosphate crystal formation combined with the systemic elevation of phosphate levels results in widespread ectopic calcifications [16], including nephrocalcinosis (Fig. 3). These inhibitors might not only act systemically but since they are also present in the kidney [76, 361, 362], they might protect from the local formation of calcium phosphate microcrystals in the tubular lumen.

## Pharmacological approaches to tackle hyperphosphatemia and hyperphosphaturia

Since hyperphosphatemia seems to drive vascular calcification and potentially various other pathologies in different tissues [16, 17], and hyperphosphaturia might contribute to kidney damage, as discussed here, pharmacological approaches to tackle the pathologic actions of phosphate should target both, hyperphosphatemia and hyperphosphaturia. However, it is challenging to achieve this goal by modulating renal phosphate uptake. For example, decreasing renal phosphate uptake does not only lower serum phosphate concentrations but also elevate tubular phosphate levels. This might be the case for some of the novel therapeutic approaches that focus on the inhibition of NaPi-2a [363]. Pfizer has developed a small molecule inhibitor for NaPi-2a which has been shown to increase urinary phosphate excretion in animals [364, 365]. In CKD patients, this approach might lower serum phosphate levels, thereby reducing pathologies associated with hyperphosphatemia, such as vascular calcification, which needs to be tested. However, at the same time NaPi-2a inhibitors might have adverse effects by increasing tubular phosphate concentrations which might cause nephrocalcinosis and nephrolithiasis thereby further damaging the kidney, as suggested by studies in NaPi-2a knockout mice [58, 135].

Vice-versa, increasing renal phosphate uptake reduces tubular phosphate levels but at the same time increases serum phosphate levels. This approach might protect from nephrocalcinosis and nephrolithiasis, while increasing serum phosphate concentrations and thereby the risk for vascular calcifications. This scenario might be the case for sodium glucose cotransporter 2 (SGLT2) inhibitors, which have potent protective effects on the cardiovascular system and the kidney [366]. SGLT2 mediates glucose uptake in proximal tubular epithelial cells, and SGLT2 inhibitors promote glycosuria thereby lowering blood glucose levels [367]. Kidney-protective effects of SGLT2 inhibitors have not only been shown in diabetes patients [366, 368–370], but also in CKD patients without diabetes [371, 372]. Furthermore, SGLT2 inhibitors seem to have only modest effects on glycemic control which seems to be insufficient to account for the breath of clinical benefits demonstrated in large clinical trials [367]. Therefore, it is likely that multiple cellular and physiologic pathways underly the beneficial actions of SGLT2 inhibitors, which includes the protection from the progression of kidney injury. Interestingly, SGLT2 inhibitors increase serum phosphate levels [373, 374]. While the underlying mechanism is not entirely clear, it is possible that SGLT2 inhibitors increase tubular phosphate uptake. Similar to NaPi-2a and NaPi-2c which cotransport phosphate and sodium, SGLT2 cotransports sodium along its concentration gradient to import glucose against its concentration gradient [375]. Therefore, it is possible that SGLT2 inhibition increases the levels of extracellular sodium that then becomes available for NaPi-2a/c-mediated phosphate transport [376]. Elevations in serum phosphate levels caused by SGLT2 inhibitors seem to be also associated with increases in FGF23 and PTH and with reductions in 1,25D [374, 377]. It is currently not known whether these induced changes in phosphate metabolism have adverse effects in SGLT2 inhibitor therapy. However, it has been reported that SGLT2 inhibitors might increase bone turnover and the risk for bone fractures [376]. Of note, while increases in serum phosphate levels seem to occur early after the initiation of SGLT2 inhibitor therapy, some studies suggest that long-term treatment actually lowers serum phosphate levels [378]. Clearly, the effects of SGLT2 inhibitors on phosphate homeostasis need to be studied in more detail, especially in patients with advanced CKD who have hyperphosphatemia, which is currently done in several clinical trials [367]. SGLT2 inhibitors might also have direct kidney protective actions by inducing hypophosphaturia. For example, it has been reported in clinical and experimental studies that SGLT2 inhibitors might prevent kidney stone formation [379–381]. While the underlying mechanism is not clear [382], this could be at least partially due to increased tubular phosphate uptake. SGLT2 inhibitors have diuretic effects [367], which also prevents stone formation. Furthermore, SGLT2 inhibitors have been shown to downregulate TLR4 expression [383], which in the proximal tubule could protect from the pathologic actions of calcium phosphate microcrystals. The potential direct kidney-protective effects of SGLT2 inhibitors need to be investigated in more detail in experimental studies.

Currently, the most promising approach to tackle hyperphosphatemia and hyperphosphaturia at the same time seems to be the reduction of dietary phosphate intake by a low phosphate diet, as discussed earlier, or by the intake of phosphate binders with the diet, which unfortunately to date has shown low efficiency in lowering serum phosphate concentrations [211–213]. Aiming to increase the levels of calcification inhibitors might be a new approach to protect CKD patients from widespread calcifications in soft tissues, including the kidney, in the presence of hyperphosphatemia and hyperphosphaturia. This could include the increase of magnesium levels via the diet or dialysate [359, 384–389] or elevating pyrophosphate levels by injections [390–392]. However, these approaches need to be targeted to the place of ectopic calcification since they should not interfere with calcifications in the bone matrix, which would have adverse effects on bone mineralization and density. 

## Conclusions

CKD is accompanied by changes in mineral metabolism, bone demineralization and calcifications in soft tissues, including the kidney. Nephrocalcinosis might not only be a consequence of CKD but further contribute to the progression of kidney damage. Calcifications in the kidney seem to include the vasculature, tubular system and parenchyma, but most of the pre-clinical and clinical studies have not provided detailed analyses of the precise localizations. More studies are needed to identify the precise areas of nephrocalcinosis, since they might differ in the cell types that are involved, the mechanisms that initiate the calcification and the consequences for kidney structure and function. Furthermore, the interconnection between calcification, tubular damage, fibrosis and inflammation in the kidney should be studied in more detail. Various pre-clinical studies provide growing evidence that phosphaturia can promote kidney injury in CKD, suggesting that tubular phosphate is a main culprit in this context. These findings have led to a novel paradigm that the cycle of high tubular phosphate causes the loss of nephrons which then further increases the phosphate load for the remaining functional nephrons and self-propagates to drive CKD progression. The underlying pathology seems to include the formation and accumulation of calcium phosphate microcrystals in the tubular lumen early during the course of kidney injury prior to increases in serum phosphate concentrations. Therefore, clinical CKD studies should not only determine serum but also urine levels of phosphate. If pre-clinical findings can be translated to the human disease, it might be beneficial to initiate phosphate lowering therapies in pre-dialysis CKD patients prior to the appearance of hyperphosphatemia. Furthermore, the precise composition of tubular calcium phosphate microcrystals, their cellular modes of action and their relevance for human CKD need to be investigated. It is possible that tubular calcium phosphate microcrystals cannot only damage the kidney but also further grow into larger crystals and eventually kidney stones with even more severe pathologic impact. Larger clinical studies are needed to determine potential associations between kidney stone formation and hyperphosphaturia, since positive outcomes would suggest that phosphate lowering therapies might be beneficial in stone formers.

While changes in phosphate metabolism are usually studied in late CKD, this article aims to raise awareness about potential phosphate abnormalities in early CKD and in various kidney diseases, including kidney stones, AKI, nephrotic syndrome, and CKDu. While for most of these diseases supporting pre-clinical data might be thin and clinical data completely missing, the appearance of hyperphosphaturia as a potential cause of kidney damage should encourage phosphate-focused studies across a wide spectrum of kidney diseases. If true, phosphate lowering therapies could be beneficial for patients with different types of kidney disease, not just ESRD patients on dialysis, which should further fuel current efforts to identify novel therapeutic targets and approaches to tackle hyperphosphaturia. Lowering dietary phosphate intake might be a valuable starting point. SGLT2 inhibitors have appeared as an exciting novel therapy for various pathologies, including kidney disease. The beneficial actions of SGLT2 inhibitors seem to go beyond their effects on glucose metabolism, and it would be interesting to determine if they include the reduction in urine phosphate levels. Hyperphosphaturia might also be a driver of kidney damage in patients with genetic diseases of phosphate imbalance, such as XLH, which needs to be determined clinically. Furthermore, since the consumption of ultra-processed food with high phosphate content is on the rise, it is of utmost importance to determine whether the chronic consumption of these diets can lead to hyperphosphaturia and kidney damage in healthy individuals, as suggested by a growing number of animal studies. Phosphate was discovered by the distillation of urine in the late 17th century, and it is up to us to determine if urine phosphate, when elevated, can damage its gate keeper.

## Data Availability

No datasets were generated or analysed during the current study.
